# Notes on the Medical Application of Electricety

**Published:** 1849

**Authors:** 


### ARTICLE VII:

*Notes on the Medical Application of Electricety.* By WILLIAM F. CHANNING, M. D. Boston: Published by Daniel Davis, Jr., and Joseph M: Wightman. 1849. pp. 100. (From Griggs, Bross, & Co., Chicago.)

This is a useful and interesting volume, devoted exclusively to the consideration of the medical properties of electricity; intended, as the author remarks, "to facilitate its application to disease."

The subject is treated under five heads, viz.:

1. Physiological Relations of Electricity.
2. Forms of Medical Electricity.
3. Means of Application.
4. General Application to Disease.
5. Special Application to Disease.

Speaking of its physiological relations, the author remarks that " the principle of vitality, in its highest signification, has hitherto escaped analysis. It is dependent, however, on physical conditions and performs its functions by the agency of physical forces." And again: " It is the product of physical organization and reacts with other physical principles."

We are aware that assertions identical with, or similar to, those just quoted are to be found in most if not all physiological works, but, as we think, without sufficient regard to their import.

If the principle of vitality has hitherto escaped analysis, what means have we of knowing that it is dependent on physical conditions, or that it performs its functions by the agency of peysical foјcs? If the principle of vitality is the product of organization, as stated above, what acts upon inorganic matter to effect that orgauization? Those who thoughtlessly make such assertions, will most certainly be driven to the absurd and impious conclusion, that matter posseses the power of self organization.

We make these remarks, not with a view of condemning the writer of the above in particular, but in order to show that broad assertions with regard to subjects not yet sufficiently investigated to justify such sweeping conclusions, are too often made, and should, therefore, be discountenanced by all lovers of truth and friends to the advancement of science.

All that portion of the work which treats of the forms of electricity, means of application, etc., is highly instructive and furnishes to the profession, in a condensed form, all the useful information upon that subject.

The numerous cases given, illustrative of its beneficial effects in the various forms of diseases in which it has been used, are interesting, and furnish indubitable evidence of its utility, and the importance of determining more certainly in what forms of disease it proves most beneficial. H.

